# Congestion of the Brain, Followed by Amnesic Aphasia

**Published:** 1887-11

**Authors:** A. H. McCord

**Affiliations:** Rusk, Texas


### CONGESTION OF THE BRAIN FOLLOWED BY AMNESIC APHASIA.

*By A. H. McCord, M. D., Rusk, Texas.*

Read before East Texas Medical Association, and by vote of Society sent for publication in Daniel's Texas Medica. Journal.

WAS called in haste on February 26, 1886, to visit Ollie C.—, age about six years, female, white, a bright and intelligent little girl. She had been "*chilling*" for several days, had one that morning at 9 o'clock; was about 1 P. M., when I arrived. She was seized with convulsion just as I entered the house, which lasted about fifteen minutes, after which there was tonic spasms of the fingers of both hands, and the forearms were flexed upon the arms, also of the muscles of the lower jaw—trismus,—with incessant clonic spasm of the muscles of the right side of the face for nearly two hours. The treatment consisted in the use of sinapisms, the entire length of the spinal column and over the epigastrium, hot baths, and cold, applied to the head. While waiting for my hypodermic syringe to come, gave per rectum, a large dose of brom. pot., tinct. digitalis, fld. ext. ergot, and chloroform by inhalation, which, in a great measure, controlled the clonic spasm of the face. One hour after, gave hypodermic dose, in arm, of 10 grs. chloral hydrat. and 8 drops tinct. digitalis; and before a sufficient interval had elapsed for another dose, the spasm and trismus were relieved so that she could swallow. The course then pursued was in the use of depressor-motors to quiet the nervous system—to prevent the return of convulsions, and to ward off a malarial paroxysm the next morning, which I succeeded in doing. Consciousness did not return for more than twenty-four hours after the convulsive seizure. When she did become gradually conscious, there was noticeable a complete amnesic aphasia, her entire vocabulary consisting of the two monosyllables, "yes," "no," indiscriminately used. There was no paralysis of any character, except memory of her former stock of articulate language. She very soon constructed a pantomime system of expressing her wants—her likes and dislikes, by movements of the hands and head, or by leading you to the object desired. Under a tonic course of treatment, she soon regained her physical health, but it was not until after the lapse of four or six months, that she had re-learned enough words to express herself in articulate language. I say "re-learned," because she was re-taught as at first—as the infant is taught. And to-day, about nineteen months since her sickness, her vocabulary is not so good as previous to that ill-fated hour.

Was the aphasia caused by a prolonged hyperæmia of the brain? or was it due to extravasation of blood in the cineritious substance of the Island of Reil? Was it uni-lateral, or bi-lateral? Or may it not have been caused by a peculiar manifestation of malaria? as she lived in the swamps of the Angelina river.

